# Interpretable prototype learning for EGFR amplification prediction from whole-slide images in glioblastoma

**DOI:** 10.1016/j.jpi.2026.100711

**Published:** 2026-08-27

**Authors:** Homay Danaei Mehr, Imran Noorani, Cong Cong, Mark Fabian, Antonio Di Ieva, Sidong Liu

**Affiliations:** a Centre for Health Informatics, Australian Institute of Health Innovation, Macquarie University, Sydney, NSW 2113, Australia; b Computational NeuroSurgery (CNS) Lab, Macquarie Medical School, Macquarie University, Sydney, NSW 2113, Australia; c Department of Neuromuscular Diseases, The Francis Crick Institute and University College London, London NW1 1AT, United Kingdom; d Department of Neurosurgery, National Hospital for Neurology and Neurosurgery, London SO16 6AQ, United Kingdom; e Department of Cellular Pathology, University Hospital Southampton NHS Foundation Trust, Southampton SO16 6YD, United Kingdom

**Keywords:** Glioblastoma, Prototype learning, Computational pathology, Whole-slide imaging, Multi-instance learning, EGFR biomarker, Histopathological image analysis

## Abstract

The epidermal growth factor receptor (EGFR) is one of the key biomarkers for diagnosis, treatment, and prognosis in glioblastoma (GBM). Current EGFR diagnostic methods, including immunohistochemistry, fluorescence in situ hybridization, and next-generation sequencing, are costly, time-consuming, and not uniformly accessible. A major challenge in whole-slide images (WSIs)-based prediction is to capture the full morphological heterogeneity of the tissue in a way that supports not only precise classification but also interpretability. To address these challenges, we proposed an automatic, interpretable prototype-learning framework that integrates a Variational Autoencoder with the Dirichlet Bayesian Gaussian Mixture Model to determine optimal tissue prototypes from morphological features of hematoxylin and eosin-stained WSI, which guide the classification of EGFR amplification. The classification performance is evaluated using internal 5-fold cross-validation on the Cancer Genome Atlas dataset and external validation on the Clinical Proteomic Tumor Analysis Consortium (CPTAC) dataset and the private GB-UK dataset. The proposed model achieved an area under the curve of 0.8087 ± 0.0153 for internal validation and 0.7740 and 0.7870 for external validations of the CPTAC and GB-UK cohorts, respectively, surpassing multi-instance learning models and conventional predefined clustering settings. Specific prototypes distinguish EGFR-amplified from EGFR-non-amplified cases, and histological analysis of these prototypes, which are consistent with expert-recognized morphological patterns, suggests an EGFR-amplified infiltration pattern. These findings demonstrate that the automatic prototype learning framework provides a rapid, cost-effective, interpretable, and scalable AI model for EGFR prediction in GBM, linking the learned prototypes to recognizable tissue patterns that can facilitate clinical decision-making.

## Highlights

• Prototype learning predicts glioblastoma-EGFR amplification from whole-slide images.
• Automatic prototype discovery captures meaningful tissue patterns.
• Automatic prototypes enhanced EGFR classification over predefined clustering.
• External validation supports generalization across external cohorts.
• Learned prototypes match expert-recognized morphological patterns.

## Introduction

Glioblastoma (GBM), one of the most aggressive intrinsic brain tumors in adults, is notoriously resistant to treatment due to its intrinsic molecular heterogeneity. The epidermal growth factor receptor (EGFR) is a protein on the surface of certain cells that plays a pivotal role in controlling cell growth, survival, proliferation, and differentiation. EGFR amplification drives the development and progression of nearly 50% of human GBMs and has been shown to initiate GBM in mouse models, making it a crucial diagnostic and prognostic biomarker, as well as a therapeutic target in GBM.^1^, ^2^, ^3^, ^4^ The 2021 WHO Classification of Central Nervous System Tumors incorporates molecular features alongside histology to provide diagnoses. The EGFR amplification serves as a diagnostic marker in isocitrate dehydrogenase1(IDH1)-wildtype GBMs, particularly in gliomas that lack histological features of aggressiveness, including microvascular proliferation and pseudopalisading necrosis.^5^, ^6^ EGFR amplification can also serve as a prognostic marker of poorer overall survival in certain populations.^7^ Hence, these findings highlight the importance of accurate identification of the EGFR amplification.

The current diagnostic methods, such as immunohistochemistry (IHC), fluorescence in situ hybridization (FISH), digital PCR,^7^ and next-generation sequencing, are costly, require technical expertise, and are time-consuming. Hence, their limited availability poses practical challenges for clinical labs. Additionally, recent biological findings indicate that EGFR amplification in GBM is typically detected in extrachromosomal DNA (ecDNA), which produces striking spatial and temporal heterogeneity in tumors, making EGFR both a prominent driver and a challenging molecule to quantify accurately.^8^

Furthermore, in recent years, significant focus has been placed on clinical trials, including those targeting GBM-EGFR amplification^9^, underscoring the critical clinical need for early, rapid, accurate, widely accessible, and scalable identification of patients with GBM-EGFR amplification, which remains a major bottleneck in the neuro-oncological clinical practice.^8^

On the other hand, AI models, especially machine learning, deep learning (DL), and modeling techniques, have demonstrated outstanding achievements in solving real-world problems. Applications have now been broadened across different fields, such as agriculture, price forecasting,^10^, ^11^ energy demand,^12^ financial and socioeconomic modeling,^13^, ^14^ and various clinical diagnoses, for instance, heart disease and breast cancer.^15^, ^16^ For each problem, a broad range of methods, such as neural networks,^17^ Gaussian process regression,^11^ and graphical causal modeling techniques,^18^ alongside different hybrid strategies, which combine with the models to capture nonlinear, heterogeneous, and independent patterns,^17^ while improving the efficiency of the predictive models.

Advancements in digital pathology, such as computerized whole-slide imaging (WSI) have enabled DL models to obtain promising results in tumor diagnosis. Weakly supervised multiple instance learning (MIL) models stand out as the state-of-the-art DL methods for WSI analysis, learning from slide-level labels rather than relying on patch-level annotations, effectively addressing challenges related to the gigantic size of WSIs, label scarcity, and the sparse distribution of tumors.^19^ Various glioma pathology studies have shown that AI-based prediction models achieved outstanding results in biomarker diagnosis from WSIs. For instance, some recent studies applied a hybrid DL-based convolutional neural network (CNN)-transformer^20^ and also MIL^21^, ^22^ models to hematoxylin and eosin (H&E)- stained WSIs to predict IDH mutation status in gliomas. Consequently, these studies provide compelling evidence that applying AI-based models to WSIs can deliver precise, scalable, and cost-effective biomarker prediction in clinical oncology.

Given the promising diagnostic results of artificial intelligence (AI) using WSIs, several studies have investigated various AI methods for detecting GBM-EGFR amplification. For the diagnosis of EGFR amplification, an AI diagnostic study^23^ has demonstrated that weakly supervised MIL models surpassed the transformer-based model used in a prior study,^24^ emphasizing the feasibility of AI methodologies in GBM-EGFR prediction. However, these AI models face severe challenges in clinical translation. Because the weakly supervised MIL approaches depend on slide-level labels and do not fully utilize unlabeled histopathology images, limit their ability to generalize across different and heterogeneous cohorts. Additionally, because WSIs contain various tissue structures that do not contribute equally to the label decision, MIL-based predictions are made on a limited set of selected WSI patches rather than the overall tissue structure. This restricts the models' ability to represent the full morphological diversity of the tissue, prevents the capture of the global tumor pattern, and makes the prediction models vulnerable to bias, providing limited insight into how distinct tissue morphologies contribute to the final prediction.

To address these challenges, we introduced an automatic and optimal prototype-based framework, integrating variational auto encoder (VAE)^25^ with Dirichlet Bayesian Gaussian Mixture Model (DBGMM)^26^ for GBM-EGFR amplification prediction directly from WSIs. Firstly, applying a VAE, which compresses the high-dimensional features of WSI-patches into a compact latent space, enables more effective clustering of tissue morphologies. Our method then employs an unsupervised prototype-learning approach that requires no annotations to discover optimal tissue morphological patterns. In fact, based on feature similarities, the model groups tissue regions into distinct morphological clusters, each represented by a prototype, known as a cluster centroid. These prototypes capture semantically meaningful tissue patterns that then guide the EGFR amplification classification model. Moreover, the significant advantage of the proposed model is that, unlike conventional unsupervised models that require a predefined number of clusters, which may lead to over- or under-clustering and affect the performance of the downstream tasks, such as classification, this model automatically determines optimal, meaningful prototypes that are desirable for more precise classification. Therefore, we aimed to integrate these prototypes into the predictive model to enhance its diagnostic performance and interpretability.

We trained our proposed model using the H&E-stained WSIs from the Cancer Genome Atlas (TCGA) EGFR dataset to determine the morphological tissue prototypes automatically. We simultaneously applied a binary classifier for predicting GBM-EGFR amplification using 5-fold cross-validation. Validation on two unseen cohorts provided encouraging evidence of the model's generalizability, although further validation on larger, multi-institutional cohorts is needed to confirm robustness in broader clinical settings. We also carried out a comprehensive performance comparison against MIL baselines^23^, ^24^, ^27^ demonstrating the high performance of our proposed model in GBM-EGFR prediction. Importantly, in this proposed model, we integrated expert pathologist interpretation of learned WSI morphologies and quantified the diagnostic contribution of each learned prototype, thereby providing a clinically interpretable view of how different regions of a tissue are assigned to semantically meaningful patterns, offering a transparent prototype distribution underlying EGFR prediction.

In summary, this prediction model, based on interpretable EGFR tissue morphologies, aims to assist pathologists in integrating it into their diagnostic workflow, thereby supporting faster, more efficient decision-making.

## Methods

The workflow of the proposed model is shown in Fig. 1. First, in the preprocessing step, we segmented the WSIs to discard the background, and then, using a tiling strategy, we divided each WSI into patches. In the next step, we extracted patch features using the UNI feature encoder, which produces 1024-dimensional patch embeddings. To compress the extracted features, we used a VAE, reducing them to 512 dimensions. These features were then processed through the DBGMM to automatically determine the optimal prototypes, which were used to cluster each WSI. The prototype-based WSI representations were then used as inputs for the slide-level classifier to discriminate between EGFR-amplified and EGFR-non-amplified cases. For fair evaluation, the compressed 512-dimensional features were fed to the MIL models.

**Fig. 1:**
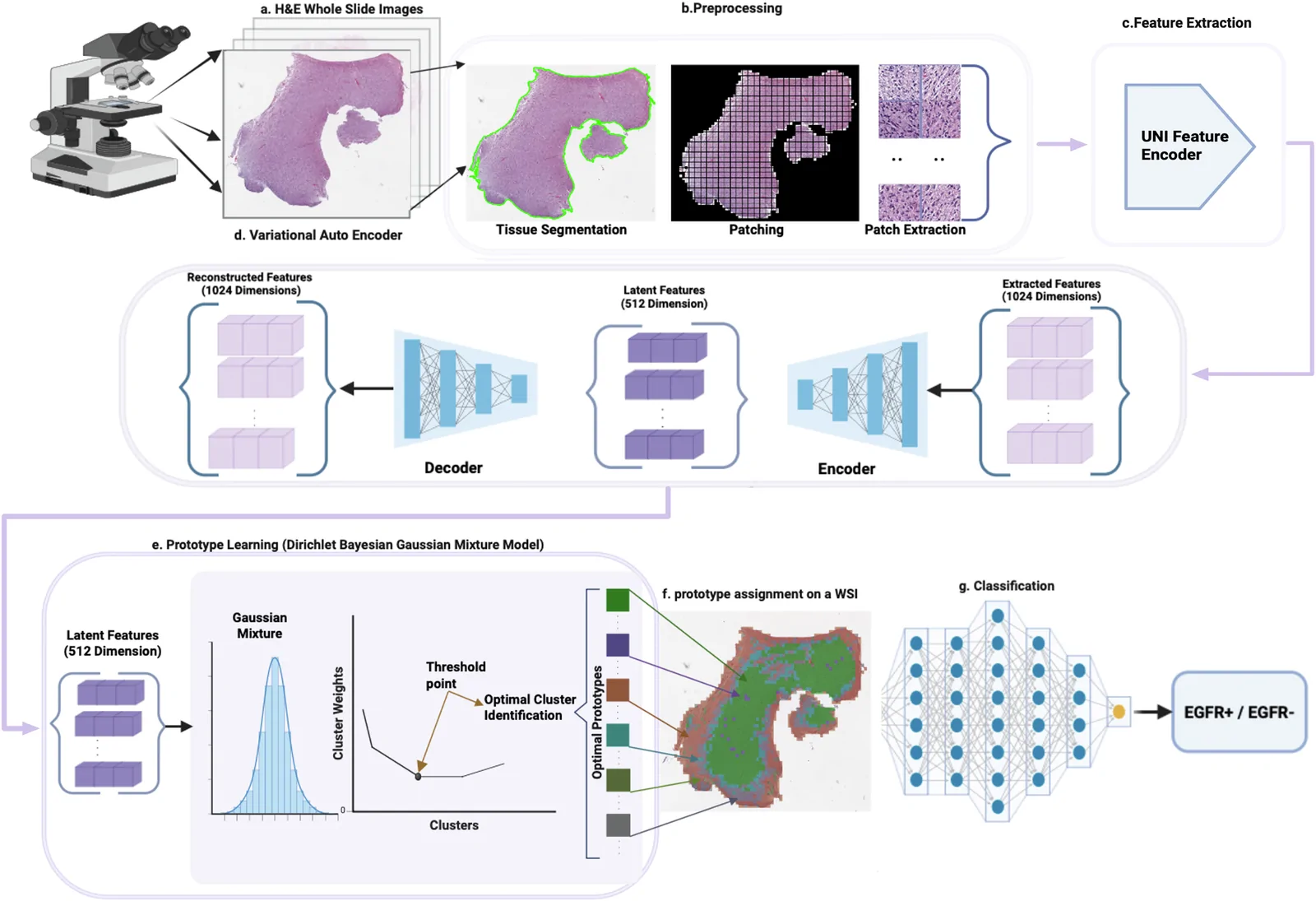
Overview of the proposed framework for EGFR classification. (a) H&E-stained WSIs. (b) WSI preprocessing, including tissue segmentation and patching. (c) Feature extraction using the UNI feature encoder which obtains 1024-dimensional patch embeddings. (d) Feature dimension compression by applying variational auto encoder (VAE), which compressed the original feature embeddings into 512-dimensional latent features. (e) Prototype learning via Dirichlet Bayesian Gaussian Mixture Model (DB-GMM), which determines the optimal clusters. The determined optimal prototypes are used to cluster each WSI, where each cluster represents the WSI's morphology. (f) Prototype assignment on a WSI, with each color corresponding to a specific prototype. (g) The prototype-based WSI representations are then utilized as inputs for the slide-level classifier to discriminate between EGFR-amplified and EGFR-non-amplified cases.

### Dataset

We utilized two public and one private GBM H&E-stained WSI datasets with EGFR amplification. The two public datasets were TCGA dataset (https://www.cancer.gov/ccg/) and the Clinical Proteomic Tumor Analysis Consortium (CPTAC) from the National Cancer Institute Centre for Cancer Genomics (https://www.cancer.gov/ccg/). The TCGA dataset comprises 329 samples, including 217 EGFR-amplified and 112 EGFR-non-amplified cases. The CPTAC dataset, used for external validation, comprises 217 samples, including 99 EGFR-amplified and 118 EGFR-non-amplified cases. EGFR amplification was defined as a copy number greater than or equal 4.^28^ Moreover, we used a private dataset (including 70 samples) from the GB-UK cohort—a single UK center,^8^ for external validation. It includes 20 WSIs with EGFR-amplified and 50 WSIs with EGFR-non-amplified cases.

### WSI preprocessing

Following the CLAM model's^29^ WSI preprocessing pipeline, we initially performed tissue segmentation^30^ and tiled the segmented tissue sections into non-overlapped smaller patches of 224 × 224 pixels using 20× magnification (Fig. 1b). Furthermore, we utilized the UNI feature encoder,^31^ a foundation model trained on over 200 million pathology H&E-stained and IHC images, to extract patch features with 1024 dimensions (Fig. 1c), which were then used in the proposed model for GBM-EGFR prediction.

### Feature compression using VAE

The VAE^25^ learns the latent representation of data using encoder and decoder components (Fig. 1d). Features can be sparse in high-dimensional data, such as WSIs, and the distances between them may become uniform. This can lead to weak clustering due to the reduction of meaningful features required for effective clustering. VAE reduces noise and data sparsity while preserving meaningful variation across tissues, minimizing the risk of overfitting in clustering.^32^, ^33^ Therefore, using the reduced and appropriate feature dimensionality results in accelerated computation tasks.^33^, ^34^

The VAE encoder learns a probabilistic mapping from high-dimensional input x to low-dimensional latent representations zapplying a variational posterior to transform high-dimensional inputs into low-dimensional representations (Eq. (1)).

(1) $$q_{\left(\emptyset \right)}\left(z|x\right)=N\left(z|\mu_{\emptyset }\left(x\right),\log\sigma_{\emptyset }^{2}\left(x\right)\right)$$

x represents the high-dimensional original patch features of a WSI, obtained during the preprocessing step. z shows the sampled latent representations, which are used by decoders during training to reconstruct the input x. The encoder predicts two vectors of the distribution, including $\mu_{\emptyset }\left(x\right)$ as the mean of latent distribution, and $\log\sigma_{\theta }^{2}\left(x\right)$ the log variance of the learned latent distribution.

The second component of the VAE is the decoder, which mirrors the encoder to map the produced low-dimensional representations back to their original high-dimensional structure. Indeed, the VAE training process is designed to iteratively adjust and refine the encoder's learned latent representations over multiple epochs through the total reconstruction loss feedback, which measures how well the decoder reconstructs the original input features x. The VAE model is optimized by minimizing the following total loss function ($L\left(x\right)$) (Eq. (2)), including reconstruction loss ($L_{\mathrm{recon}})$ and Kullback–Leibler divergence loss ($l_{\mathrm{kl}})$.

(2) $$L\left(x\right)=L_{\mathrm{recon}}+L_{\mathrm{kl}}$$

(3) $$l_{\mathrm{recon}}={\left\|\;x-\hat{x}\;\right\|}^{2}$$

$l_{\mathrm{recon}}\;$is a mean-squared error (MSE) (Eq. (3)), which calculates the loss between the input x and the reconstructed input $\hat{x}$ and the $L_{\mathrm{kl}}$ (Eq. (4)) measures how well the latent space aligns with the standard normal prior $N\left(0,I\right)$, where I is the identity matrix, d is the dimension of the latent presentations, and $\mu_{i}$ represents ith element of the mean vector $\mu_{i}\left(x\right)$ produced by the encoder. Moreover, $\sigma_{i}^{2}$ indicates ith element predicted variance vector.

(4) $$L_{\mathrm{kl}}=-\frac{1}{d}\sum_{i=1}^{d}\left(1+\log\sigma_{i}^{2}-\mu_{i}^{2}-\sigma_{i}^{2}\right)$$

### Unsupervised optimal prototype discovery with DBGMM

In contrast to the majority of conventional clustering methods, which require predefined numbers of clusters, the DB-GMM takes advantage of the flexibility of the Dirichlet process (DP)^35^ and the Gaussian Mixture Models (GMMs)^36^ to determine the optimal clusters in a data-driven process.

The DP is a Bayesian nonparametric approach used to model distributions to learn the number of clusters based on infinite mixture of clusters. DP is defined in Eq. (5), where α indicates the concentration parameter that determines the likelihood of possible new clusters. As the value of α increases, it permits more clusters. $G_{0}$ presents the base data distribution over cluster parameters such as the component mean.

(5) $$\mathrm{DP}\left(\alpha ,G_{0}\right)$$

In contrast to GMM,^36^ DBGMM does not rely on a fixed number of clusters and incorporates a non-parametric Bayesian prior in clustering. It fits the input data (latent vectors) $\left(z\right)$ using the following (Eq. (6)):

(6) $$P\left(z|w,\theta \right)=\sum_{k=1}^{K}w_{k}N\left(z|\mu_{k},\sum_{k}\right)$$

where $N\left(z|\mu_{k}\sum_{k}\right)$ represents the Gaussian component with mean ($\mu_{k})$ and covariance $\left(\sum_{k}\right)$. K is the number of components, and the DP assigns clusters. $w_{k}$ is the weight of cluster *k* derived from the DP.

The latent representations are assigned to Gaussian components with different probabilities according to their posterior responsibilities, which are derived from each Gaussian component's likelihood in the Gaussian distribution with its prior weight. Consequently, through a Bayesian prior, components receive low posterior weights if they contain a minimum number of assigned data (latent vectors). To refine the selection of meaningful clusters, we utilized a data-driven thresholding procedure based on the Elbow method^33^ applied to the posterior weights of the components to identify a weight threshold to prune low-weight components. To reduce the fluctuations in weight distribution and produce a stable curve for knee point detection, the Gaussian smoothing^37^ was applied to the posterior cluster weights. Considering w as the sorted posterior weights $w=\left[w_{1},w_{2},\ldots ,w_{K}\right]\;$), of the K components derived from DBGMM, the Gaussian smoothing of these weights was calculated as Eq. (7). $\text{GaussianFilter}1D\left(.,\sigma \right)$ denotes one-dimensional convolution with a Gaussian kernel of standard deviation (σ)*.*

(7) $$\tilde{w}=\text{GaussianFilter}1D\left(w,\sigma \right)$$

Eq. (8) indicates how smoothed values are computed. i denotes the rank position being smoothed, whereas j ranges over all rank positions in w. $w\left(j\right)$ shows unsmoothed/raw weights at rank j and Z ensures the sum of all j results in 1. The rank distance ∣i−j∣, which shows how many positions apart two components are in the sorted sequence. Components with smaller ∣i−j∣, ranked close to i, are assigned larger kernel weight and contribute more strongly to $\tilde{w}\left(i\right)$. This contribution decreases according to a Gaussian envelope whose width is set by σ.

(8) $$\tilde{w}\left(i\right)=\frac{1}{Z}\sum_{j}w\left(j\right)e^{-\frac{{\left(i-j\right)}^{2}}{2\sigma^{2}}},Z=\sum_{j}e^{-\frac{{\left(i-j\right)}^{2}}{2\sigma^{2}}}$$

Here, instead of manually tuning a fixed σ, it is adaptively selected based on the variability in the weight distribution itself, as given in Eq. (9). In fact, σ denotes the degree of smoothing applied to the sorted component weight curve. Therefore, a higher value permits comparatively more smoothing. In contrast, the curve is moderately smoothed with a smaller value of $\mathrm{Std}\left(w\right)$.

(9) $$\sigma =\mathrm{Std}\;\left(w\right)$$

More generalized σ is presented by $\sigma =c\cdot \mathrm{Std}\left(w\right)$, where c is the scale factor. Its variation tests the robustness of the σ in smoothing the weight curve. In this study, c is set to 1, and for the sensitivity analysis, we applied different values of it. Following the smoothing process, the Elbow method was applied to the cumulative sum of the smoothed weights. In this study, the Elbow method was not applied to directly select the number of clusters; rather, it was used to identify a weight threshold to prune low-weight components in DBGMM. Indeed, the Elbow method identifies the inflection point (knee point), which corresponds to the maximum curvature at rank position $i^{*}$. The unsmoothed weight corresponding to the inflection point (weight_threshold = $w\left(i^{*}\right)$) is the data-driven weight threshold. Components with weights ($w_{k})\;$exceeding this threshold were retained, whereas components below the threshold were discarded (Eq. (10)). The retained clusters and their corresponding centroids serve as the final WSI prototypes for downstream tasks (Fig. 1e).

(10) $$\mathrm{Cluster}=\left\{\;\mu_{k}|w_{k}\geq \mathrm{weight}\_\mathrm{threshold}\right\}$$

### GBM-EGFR classification

The optimal prototypes identified by our proposed model, or the predefined prototypes used in competing models, were used for the downstream classification task. For classification purposes, each WSI using its patch features was mapped onto this prototype space (Fig. 1f). Then, a WSI-specific mixture proportion, mean vector, and covariance vector were calculated for each prototype, and then protype-specific statistics were combined to make a WSI-specific representation. In fact, for classification, these slide representations were separated into set of prototype-specific vectors. The count of vectors is equal to the count of prototypes. Therefore, for each vector, an independent MLP was applied, allowing the model to learn distinct morphological patterns. The produced prototype embeddings were then concatenated and passed through a final linear layer^38^ that generated two class logits for the EGFR-amplified and EGFR-non-amplified class labels.

Furthermore, because MIL-based weakly supervised learning models have gained popularity for their ability to leverage slide-level annotations,^39^ in this study, three state-of-the-art MIL models, including CLAM,^29^ TransMIL^40^, DTFD,^41^ and attention challenging MIL (ACMIL)^42^ were used for comparison purposes. CLAM is an attention-based MIL model, whereas DTFD is a two-stage strategy model that uses attention and feature distillation to classify WSIs. Moreover, TransMIL is a transformer-based model that utilizes self-attention to classify WSIs, whereas ACMIL is a multi-attention model that applies Top-k instance masking to hide the highest-attention instances, forcing the model to learn from more challenging patches. Therefore, we used slide-level classification rather than computationally expensive and labor-intensive patch-level predictions.

### Evaluation metrics and statistical methods

The classification models were trained using 5-fold cross-validation on the TCGA dataset (329 samples), with a split of 80% for training, 10% for internal validation, and 10% for testing in each fold. The 5-fold cross-validation results are reported as the mean and standard deviation of the performance metrics. For external validation, we assigned 85% of the TCGA dataset for training and 15% for internal validation. Subsequently, we allocated the entire CPTAC (217 samples) and GB-UK (70 samples) datasets as unseen test sets. All splits were at the patient level. The area under the curve (AUC), sensitivity (true-positive rate), specificity (true-negative rate), and F1-score were used to assess performance. To quantify each prototype's contribution to the model's decision-making, we evaluated the distribution using Cohen's d relative to each prototype's probability. Cohen's d effect size was computed using the mean prototype probabilities based on the WSIs' ground-truth, EGFR-amplified and EGFR-non-amplified labels, scaled by the pooled standard deviation.

### Implementation details

#### VAE

Following the preprocessing step of WSIs, the extracted features of each WSI goes through the VAE framework step. First, the encoder projects the 1024-dimensional input using two hidden layers with monotonically decreasing width (1024 → 768 → 640). Afterwards, it branches into two parallel linear output layers of 640 → 512, each independently providing the $\mu_{\phi }\left(x\right)$and $\log\sigma_{\phi }^{2}\left(x\right)$. Then, the decoder mirrors these steps and reconstructs the 1024-dimensional data with the final linear output layer. In each hidden layer, a linear transformation followed by normalization, ReLU nonlinearity, and a dropout rate = 0.2 was applied. Due to the computational simplicity of ReLU and its avoidance of vanishing gradients, which are prevalent issues in tanh and sigmoid, we selected ReLU for hidden layers. Kaiming^43^ initialization is used for hidden-layer weights, which is consistent with ReLU, whereas Xavier initialization^44^ was applied to the posterior parameter heads, ($\mu_{\phi }\left(x\right)$ and $\log\sigma_{\phi }^{2}\left(x\right)$ and the final reconstruction layer. Because gradual dimension reduction prevents the loss of information,^45^ we applied this dimension reduction strategy rather than a single direct reduction. For 1024 → 768 → 640 → 512, using a similar compression ratio at each step: ∼0.75, ∼0.83, ∼0.80, avoids one layer being disproportionately more compressed than others. The VAE was optimized by minimizing the total loss utilizing Adam with an initial learning rate of 0.001 and no additional weigh decay. The learning rate was reduced by a factor of 0.5. Training was conducted for a maximum of 50 epochs. Based on the devised early stopping, the training was stopped early if the monitored validation failed to improve by at least 0.0001 for 10 consecutive epochs. Moreover, a batch size of 64 was preferred. We utilized cloud-based computational resources, including a NVIDIA V100 GPU with 64 GB of GPU memory and 64 GB of system memory. Moreover, to ensure a fair evaluation, we applied VAE to all three datasets.

#### DBGMM

DBGMM was applied on whole TCGA cohort using scikit-learn's Bayesian Gaussian Mixture implementation with the following configurations. The K components were assigned to 50 with diagonal covariance ($\sum_{k})$. The DP concentration parameter was assigned to α=0.05. Other priors were kept as their standard bayes defaults. The default standard conjugates Normal-Wishart (Normal-Gamma under diagonal covariance) was taken for $G_{0}$. Regarding convergence criteria, the model was fit up to 500 variational EM iterations alongside a convergence tolerance of 0.001. Before knee-point detection, the sorted component weights were smoothed using $\sigma =\mathrm{Std}\left(w\right)$ with a scale factor (c=1). Robustness of the retained prototype set was evaluated through different scaling factors in the ablation study section.

#### WSI classification

The downstream classification model used an individual MLP for each prototype-specific representation, which was six optimal prototypes for our proposed framework and different numbers for competing models. Each MLP included a single fully connected layer followed by a ReLU activation. These resulting prototype embeddings were then concatenated and passed through a final linear layer to produce EGFR-amplified and EGFR-non-amplified class logits. The classifier was trained utilizing cross-entropy loss and the Adam optimizer with a learning rate of 0.0001 and dropout of 0.1. We trained the model for a maximum of 200 epochs, with early stopping when no improvement was observed for 30 consecutive epochs. Furthermore, the model was trained with a fixed random seed.

## Results

### Prototype learning discovers distinct morphological prototypes in glioblastoma

Following the EGFR WSI dataset preprocessing (Fig. 1b), 1024-dimensional patch features were extracted (Fig. 1c). We trained the classification model with different compressed dimensional sizes. Hence, the application of VAE-compressed 1024-dimensional features to 512 low-dimensional latent representations (Fig. 1d).

We applied DBGMM (Fig. 1e) to identify an optimal set of morphological prototypes from 512-dimensional features, yielding six distinct prototypes. These distinctive and optimal prototypes captured semantically meaningful tissue patterns without requiring predefined cluster numbers. Fig. 2(a1) and (b1) are examples EGFR status illustrating the original H&E-stained WSI, and the optimal prototype assignment maps over a representative WSI. Each patch is assigned to one of six defined clusters, showing spatial heterogeneity captured in the feature space. Furthermore, Fig. 2 (c1) (Proportion-Cluster) depicts the estimated prototype distribution within a WSI. In fact, it quantifies the proportion of patches per prototype, showing that the relative abundance of prototypes varies significantly across WSIs. Each bar corresponds to one of the six optimal clusters (C0–C5) determined by the proposed model. For instance, for a1 (Proportion-Cluster distribution) cluster C4 was the dominant tissue section, followed by cluster C5, and other clusters represent a lower proportion of the WSI. Moreover, as shown in Fig. 2c1, for b, the Proportion-Cluster distribution indicated that C3 was the dominant prototype, whereas C2 contributed a moderate proportion. Other clusters' proportion contribution remained at a minimal level. Fig. 2(c2) illustrates representative patch examples for each prototype in each WSI. The patches of each prototype were reviewed by a neuropathologist, who provided morphological interpretation. Therefore, these descriptions provide more clinically and morphologically interpretable prototype patterns.

**Fig. 2:**
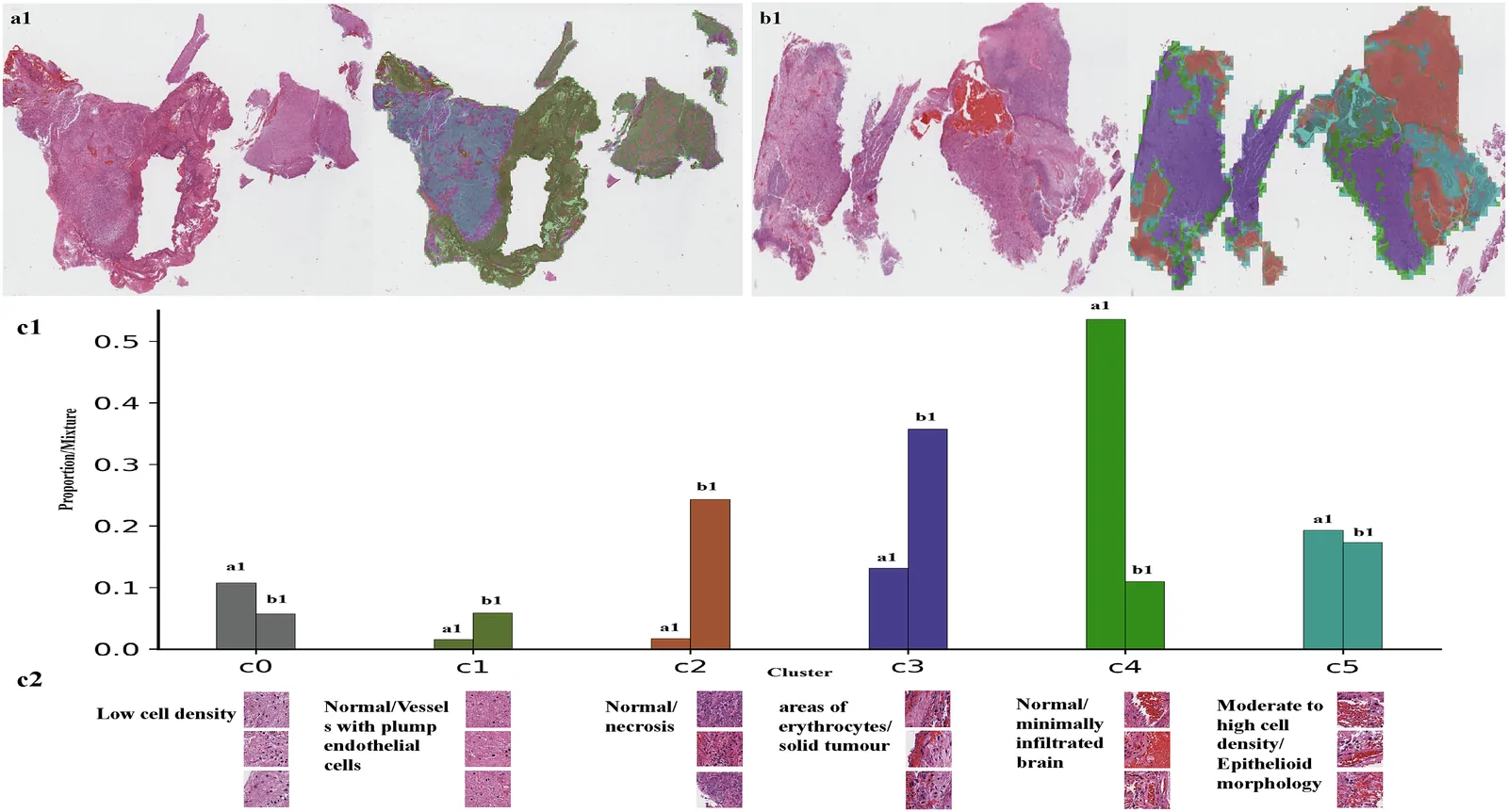
Visualization of the optimal prototypes derived by the automatic prototype learning framework on a representative WSI. (a1) and (b1) illustrate the original H&E-stained WSIs and the spatial distribution of patch-level cluster assignments on WSI tissues. (c1) Proportional visualization of patches assigned to each of the six optimal prototypes. Cluster 4 (C4) exhibits the highest mixture proportion, followed by a moderate proportion of patches by cluster 5 (C5) in a1. In b1, cluster 4 (C4) has the most extensive tissue coverage, followed by cluster 2 (C2) with a moderate proportion contribution. c2 panels show the representative patches related to each prototype, illustrating the distinct morphological patterns identified within each WSI. The reviewing neuropathologist added brief morphological descriptions after the model identified the prototypes.

### Prototype-specific contributions to EGFR amplification prediction

We examined the distribution of prototypes based on the proportion of patches assigned to each of the six optimal prototypes across the test set (Fig. 4). Although the relative distribution of prototypes varied across WSIs (Fig. 4), the morphological prevalence of the prototypes did not reflect their diagnostic contribution. Therefore, to quantify the contribution of each learned morphological prototype to the EGFR amplification prediction, we evaluated the distribution using Cohen's d relative to prototype probabilities. To clarify how we derived each prototype-specific probability of each WSI, following the classification section, once the six optimal prototypes were identified, the mean and covariance vectors for each prototype were computed for each WSI. These prototype-specific statistics were combined to form a WSI-specific representation; subsequently, they were reorganized into six vectors, and each vector went through a prototype-specific MLP. The resulting prototype embeddings were concatenated and passed through a final linear layer, generating two class logits. To extract the prototype-specific label probability, the weight matrix of the final linear layer was divided into six weight blocks, each corresponding to one of the six concatenated prototype embeddings. Each weight block was applied to its corresponding prototype embedding to derive two prototype-specific logit contributions, representing support for the EGFR-amplified and EGFR-non-amplified classes. Softmax was then applied separately to each prototype-specific logit pair, producing prototype-specific class-support probabilities. As a result, for six prototypes, each WSI yielded six probability pairs, where each prototype-specific probability shows the class support provided by that prototype in the classification of the given WSI. These prototype probabilities, which are uncalibrated class supports specific to prototypes, were used only in this section for Cohen's d calculation. They are not six separate final WSI prediction results. The primary overall classification outcomes in this study are derived from the combined logit contributions of all prototypes, with the final linear layer generating two class logits. Therefore, Table 1 shows the mean prediction probability for each of the six optimal prototypes separately for WSIs with EGFR-amplified and for WSIs with EGFR-non-amplified labels. On average, across the test slides shown in Table 1, prototypes C4, C3, C2, and C5 had higher mean probabilities in WSIs with EGFR-amplified labels, indicating more support for an EGFR-amplified diagnosis. Conversely, prototypes C1 and C0 showed higher probabilities for EGFR-non-amplified labels. Furthermore, Cohen's d was computed considering the derived prototype-specific class support probabilities.

**Table 1:** Prototype-specific contribution patterns for EGFR-amplified and EGFR-non-amplified.

Prototype	Mean probability in WSIs with EGFR-amplified label	Mean probability in WSIs with EGFR-non-amplified label	Cohen's d
C0	0.5732	0.4507	0.7326
C1	0.5389	0.4903	0.3269
C2	0.6268	0.4507	1.0589
C3	0.6491	0.4394	1.0463
C4	0.7048	0.3616	2.0790
C5	0.6050	0.4064	1.1575

Cohen's d was calculated using the difference between the mean probabilities of these two groups, standardized by their pooled standard deviation, thereby measuring differences in prototype-specific correct-class confidence between EGFR-amplified and EGFR-non-amplified cases. Indeed, Cohen's d quantifies how strongly each prototype separates WSIs with EGFR-amplified labels from EGFR-non-amplified labels. Hence, Cohen's d was calculated as the difference between the mean of all prototype-specific probabilities supporting the WSIs' ground-truth for EGFR-amplified and EGFR-non-amplified labels, divided by the pooled standard deviation. According to Table 1, the effect size was large for prototype C4, with a very large Cohen's d (2.0790), indicating a robust separation between EGFR-amplified and EGFR-non-amplified. This was closely followed by prototypes C5 (Cohen's d = 1.1575), C2 (Cohen's d = 1.0589), and C3 (Cohen's d = 1.0463), which had a high discriminative impact on the model's EGFR-amplified decision. Conversely, C0 presented a medium effect size at Cohen's d = 0.78, whereas C1 had a small effect size (Cohen's d = 0.39).

This effect size quantifies separation in model-driven prototype scores and does not directly interpret the prototypes' corresponding morphological description quality specific to EGFR amplification prediction. Nevertheless, considering the strong separation of C4 and C3 and the expert's histopathological review of the slides revealed that C4 and C3 prototypes were characterized by low-cell-density regions with either minimally infiltrated or subtly abnormal brain tissue (Fig. 2), suggestive of a distinct EGFR-amplified infiltration pattern.

### Optimal clusters outperform predefined clustering settings in EGFR amplification prediction

We conducted a comprehensive classification evaluation based on automatically determined clusters (six optimal clusters from the proposed automatic prototype learning) and manually selected predefined cluster numbers, followed the PANTHER^38^ framework (using k-means clustering strategy). Then, these prototypes were used in the classification stage. Table 2 summarizes MLP classification results for the proposed automatic prototype learning-based prototypes and predefined prototype numbers, obtained via 5-fold cross-validation on the TCGA cohort comprising 329 samples.

**Table 2:** 5-fold cross-validation classification results (on TCGA cohort) based on clustering methods and given prototype numbers.

Clustering	Prototype	AUC (Mean ± SD)	Specificity (Mean ± SD)	Sensitivity (Mean ± SD)	F1-score (Mean ± SD)	*p*-value (*t*-test)	p-value (Wilxon)
Proposed automatic prototype learning	**6**	**0.8087** **± 0.0153**	**0.7380** **± 0.1181**	**0.9385** **± 0.0422**	**0.9066** **± 0.0326**	–	–
Predefined cluster numbers	4	0.6914 ± 0.0402	0.4444 ± 0.0314	**1.0000 ± 0.000**	**0.8780** **± 0.0229**	**0.0062**	**6.828*E*-07**
	5	0.7408 ± 0.0230	0.4775 ± 0.1407	0.9900 ± 0.0200	0.8597 ± 0.0844	**0.0075**	**1.30E-06**
	6	0.7653 ± 0.0172	**0.5654 ± 0.0560**	0.9900 ± 0.0200	0.8760 ± 0.0706	**0.0023**	**1.48E-06**
	**7**	**0.7746 ± 0.0352**	0.5360 ± 0.1171	**0.9900 ± 0.0200**	0.8696 ± 0.0846	**0.0344**	**1.30E-06**
	8	0.7568 ± 0.0418	0.1600 ± 0.2585	0.9895 ± 0.0211	0.7954 ± 0.1240	0.067	**3.51E-06**
	9	0.7544 ± 0.0218	0.5137 ± 0.0994	0.9900 ± 0.0200	0.8650 ± 0.0821	**0.0038**	**1.30E-06**
	10	0.7508 ± 0.0143	0.4985 ± 0.1415	0.9900 ± 0.0200	0.8660 ± 0.0793	**0.0045**	**1.30E-06**
	11	0.7467 ± 0.0251	0.5061 ± 0.1101	0.9835 ± 0.0209	0.8592 ± 0.0841	**0.0022**	**1.20E-06**
	12	0.7452 ± 0.0277	0.4929 ± 0.1293	0.9900 ± 0.0200	0.8648 ± 0.0749	**0.0093**	**1.45E-06**
	13	0.7385 ± 0.0207	0.4399 ± 0.1504	0.9900 ± 0.0200	0.8505 ± 0.0916	**0.0036**	**1.16E-06**
	14	0.7370 ± 0.0202	0.4419 ± 0.1302	0.9900 ± 0.0200	0.8516 ± 0.0836	**0.0028**	**1.20E-06**
	15	0.7289 ± 0.0196	0.4553 ± 0.1353	0.9900 ± 0.0200	0.8553 ± 0.0828	**0.003**	**1.30E-06**
	16	0.7112 ± 0.0423	0.0133 ± 0.0267	**1.0000** **± 0.000**	0.7810 ± 0.1050	**0.0026**	**9.43E-07**
	17	0.7074 ± 0.0217	0.4086 ± 0.0800	0.9900 ± 0.0200	0.8472 ± 0.0800	**0.0008**	**9.13E-07**
	18	0.6817 ± 0.0425	0.0133 ± 0.0267	**1.0000** **± 0.000**	0.7810 ± 0.1050	**0.0036**	**1.14E-07**
	32	0.6255 ± 0.0636	0.1133 ± 0.1950	**1.0000** **± 0.000**	0.7931 ± 0.1173	**0.0001**	**8.41E-08**

In **Table 2**, boldface highlights the proposed model results and the highest numerical values among the predefined-prototype competing models for each performance metric; bold p-values indicate statistically significant differences at *p* < 0.05. These highlighted values are interpreted in the Results section

The results demonstrated that the classifier using 6 optimal prototypes outperformed (AUC: 0.8087 ± 0.0153 and F1-score: 0.9066 ± 0.0326) all classification results based on the k-means clustering settings with various cluster numbers, which achieved the highest performance at seven clusters (AUC = 0.7746 ± 0.0352, F1-score = 0.8696). However, these results are still lower than the classifier using the optimal prototypes automatically derived from the proposed model. Furthermore, although several classification models using k-means-derived prototypes (e.g., 4–15 prototypes) achieved high F1-scores (>0.85), they consistently showed high sensitivity but comparatively low specificity, indicating a tendency to over-predict EGFR-amplified classes. In contrast, the classifier using optimal prototypes, achieved a higher AUC with a more balanced sensitivity and specificity. The receiver operating characteristic (ROC) curves (Fig. 5a) show that the classifier using the optimal clusters achieved a higher AUC for predicting EGFR cases. Fig. 5b illustrates the variation in classification performance (AUC) of the k-means-derived prototypes. The curve shows that AUC initially increases, reaches a peak at seven clusters (AUC of (0.7746 ± 0.0352)), and then declines, indicating diminished classification performance with large numbers of clusters. These findings underscore the diagnostic importance of automatically learning the representative tissue morphologies, achieved by our proposed model, in supporting more accurate GBM-EGFR classification from H&E-stained WSIs.

To investigate whether the proposed model's AUC improvements over 16 baselines were statistically significant, we conducted two statistical analyses (Table 2). First, a paired *t*-test was applied to fold-level AUC values from the 5-fold cross-validation splits comparing the proposed model against each predefined-prototype baseline. The results demonstrate that the improvement was statistically significant for 15 out of 16 models, with *p*-values <0.05. The baseline model with eight predefined prototypes showed a positive AUC difference; however, it did not reach statistical significance, suggesting that reliance on 5-fold cross-validation constrains statistical robustness, particularly when fold-level comparisons are smaller or less consistent across folds. Furthermore, we conducted a second statistical analysis: a one-sided paired Wilcoxon signed-rank test using the predicted probability assigned to the ground-truth labels. The results showed *p*-values ranging from $8.41\times 10^{-8}$ to $3.51\times 10^{-6}$, demonstrating the proposed model's significance over 16 baseline models (*p*-value <0.05). To further examine classification errors, representative false-negative (FN) and false-positive (FP) cases, along with their corresponding prototype distributions, are shown in Fig. 3. Across the 5-fold cross-validation on TCGA, 14 FP and 7 FN predictions were observed, yielding a mean overall error rate of 11.88% ± 2.95%.

**Fig. 3:**
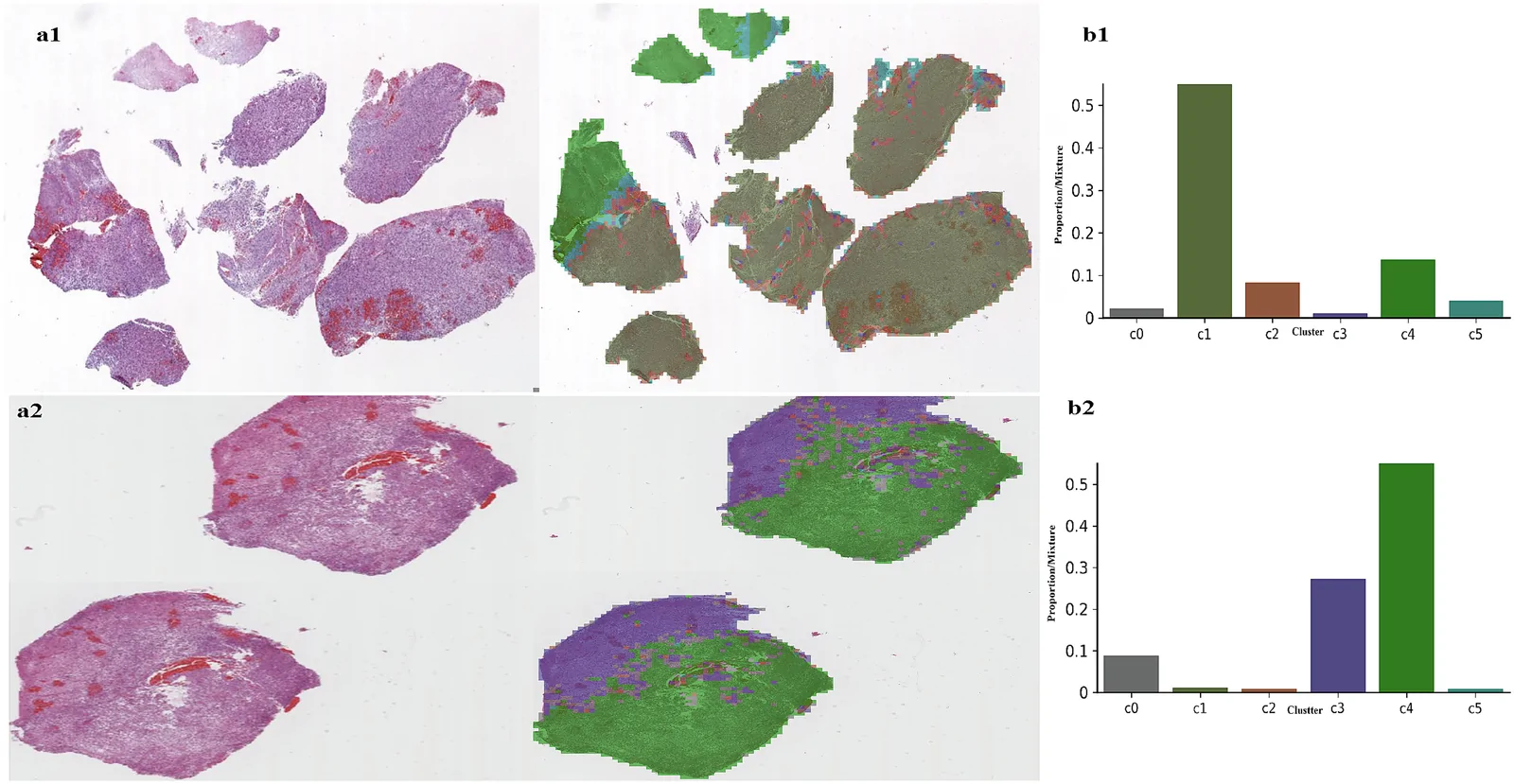
Visualization of misclassified cases on representative WSIs. (a1) and (a2) illustrate the original H&E-stained WSIs and the spatial distribution of patch-level cluster assignments on WSI tissues for FN and FP cases, respectively. (b1 and b2) Proportional visualization of patches assigned to each of the 6 optimal prototypes for FN and FP cases, respectively.

**Fig. 4:**
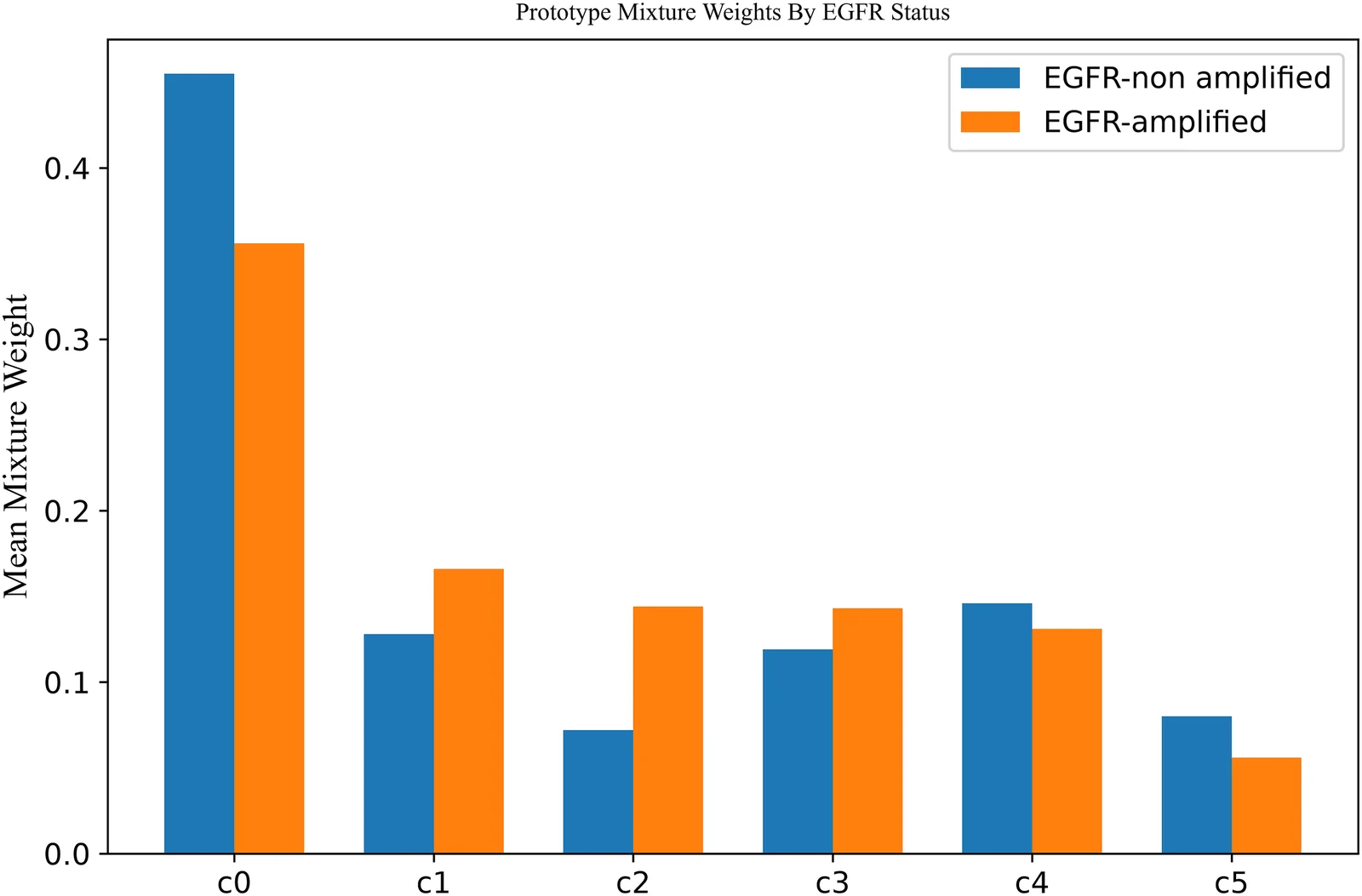
Aggregated prototype mixture weights of EGFR status. The plot shows the mean proportion of patches assigned to each of the six optimal prototypes across EGFR-non-amplified and EGFR-amplified WSIs.

**Fig. 5:**
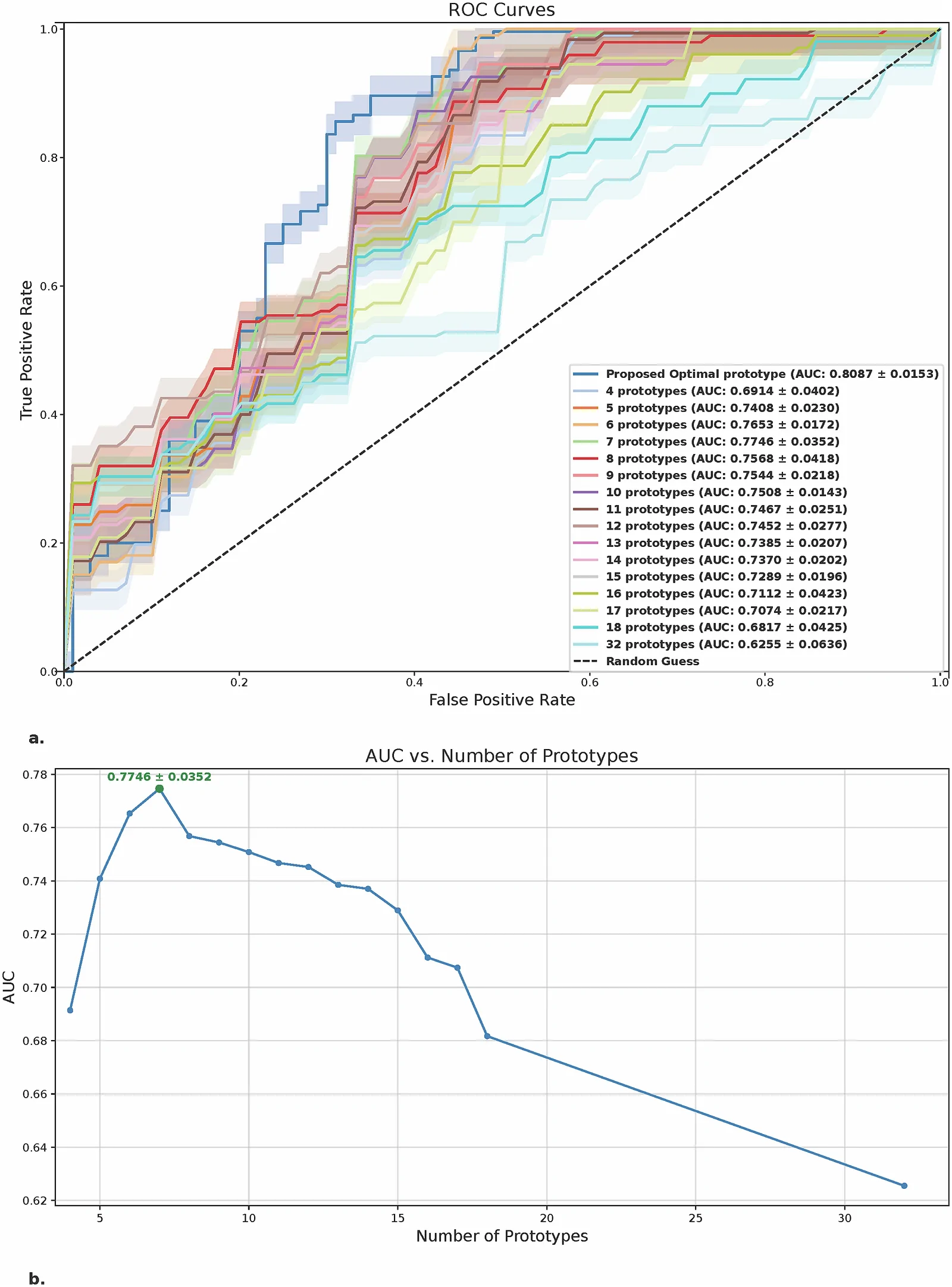
EGFR classification performance evaluation across various prototype settings. (a) ROC curves were attained from a 5-fold cross-validation setting using the TCGA dataset. The MLP classifier employing the optimal six prototypes derived from the proposed model obtained a superior AUC of 0.8087 ± 0.0153, outperforming the MLP classifiers using predefined prototypes. (b) Illustration of the relationship between AUC of the EGFR classification and the number of prespecified clusters based on k-means. The highest performance was observed at seven prespecified clusters (AUC:0.7746 ± 0.0352), followed by a consistent AUC degradation, suggesting model's degradation related to over-clustering.

### EGFR amplification prediction using optimal clusters generalizes across cohorts

Considering the importance of developing a GBM-EGFR diagnostic model that generalizes across various cohorts, we conducted a comprehensive evaluation of our proposed optimal clustering-based classification model using two unseen external cohorts: CPTAC (including 217 samples) and GB-UK (including 70 samples). The classifier utilized the TCGA dataset (including 329 samples) as the training set and the two unseen datasets as external test sets. Subsequently, the model was trained using the proposed automatic learning-derived optimal clusters, as well as a series of predefined cluster numbers (4–18 and 32), based on the k-means. Table 3 and Table 4 summarize the classification results of the CPTAC and GB-UK evaluations, respectively.

**Table 3:** Classification performance evaluation using the TCGA trainset and the CPTAC unseen test set.

Clustering	Prototype	AUC	Specificity	Sensitivity	F1-score
Proposed automatic prototype learning	**6**	**0.7740**	**0.6525**	**0.8585**	**0.7555**
Predefined cluster numbers	4	0.7119	0.5084	0.9494	0.7490
	5	0.7165	0.5338	0.9494	0.7580
	6	0.7262	0.5423	0.9494	0.7611
	**7**	**0.7312**	**0.5508**	0.9494	**0.7642**
	8	0.6970	0.1300	**1.0000**	0.786
	9	0.6916	0.4661	0.9494	0.7343
	10	0.6899	0.4745	0.9393	0.7322
	11	0.6896	0.4576	0.9393	0.7265
	12	0.6881	0.4745	0.9292	0.7272
	13	0.6864	0.4491	0.9494	0.7286
	14	0.6797	0.4745	0.9191	0.7222
	15	0.6785	0.4661	0.9191	0.7193
	16	0.6670	0.4290	**1.0000**	0.750
	17	0.6644	0.4067	0.9393	0.7099
	18	0.6610	0.5710	0.8780	0.7780
	32	0.6400	0.355	**1.0000**	0.723

In **Tables 3 and 4**, boldface highlights the proposed model results and the highest numerical values among the predefined-prototype models for each metric, including individual high values that are specifically interpreted in the Results section in the context of the other performance measures. It is interpreted in the Results section.

**Table 4:** Classification performance evaluation using the TCGA trainset and GB-UK unseen test set.

Clustering	Prototype	AUC	Specificity	Sensitivity	F1-score
Proposed automatic prototype learning	**6**	**0.7870**	**0.660**	**0.90**	**0.6546**
Predefined cluster numbers	4	0.7241	0.5423	0.9494	0.7611
	5	0.7372	0.5593	0.9494	0.7673
	6	0.7488	0.5762	0.9494	0.7736
	7	**0.7551**	**0.5847**	0.9494	**0.7768**
	8	0.7430	0.540	0.950	0.6129
	9	0.7410	0.5677	0.9494	0.7704
	10	0.7312	0.5508	0.9494	0.7642
	11	0.7217	0.5423	0.9494	0.7611
	12	0.7179	0.5338	0.9494	0.7580
	13	0.7158	0.5254	0.9494	0.7550
	14	0.7138	0.5254	0.9393	0.750
	15	0.7119	0.5084	0.9494	0.7490
	16	0.7067	0.5169	0.9393	0.7469
	17	0.6928	0.4745	0.9494	0.7372
	18	0.690	0.360	**1.0000**	0.555
	32	0.6350	0.2800	**1.0000**	0.5263

In **Tables 3 and 4**, boldface highlights the proposed model results and the highest numerical values among the predefined-prototype models for each metric, including individual high values that are specifically interpreted in the Results section in the context of the other performance measures. It is interpreted in the Results section.

Using optimal clusters, the classifier applied to the unseen CPTAC dataset (Table 3) yielded an AUC of 0.7740, specificity of 0.6525, sensitivity of 0.8585, and F1-score of 0.7555, surpassing all classifiers using predefined k-means-derived prototypes. The highest performance observed among prespecified cluster numbers was achieved by the classifier utilizing seven clusters with an AUC of 0.7312. However, classification performance diminished as the number of predefined clusters exceeded this peak. For example, when the cluster numbers increased to 32, overall performance decreased significantly, with AUC dropping to 0.6400, specificity to 0.355, and F1-score to 0.723, despite sensitivity remaining at 1. These findings highlight the negative impact of over-clustering, which results in an artificial increase in sensitivity at the cost of specificity and diminishes the classifier's performance in distinguishing between EGFR-amplified and EGFR-non-amplified cases.

Fig. 6a illustrates the ROC curves of the classifiers using clusters derived from our proposed model versus predefined settings on the CPTAC test set. The high performance of the classifier based on optimal clusters is visually noticeable, emphasizing the generalizability of using automatic prototype learning-derived clusters in classifying EGFR mutation while utilizing unseen and external datasets. Furthermore, Fig. 6b depicts the AUC trends for the classifiers across various prespecified cluster numbers. The classifier achieved its highest performance using seven clusters, after which the performance declined as the number of clusters increased.

**Fig. 6:**
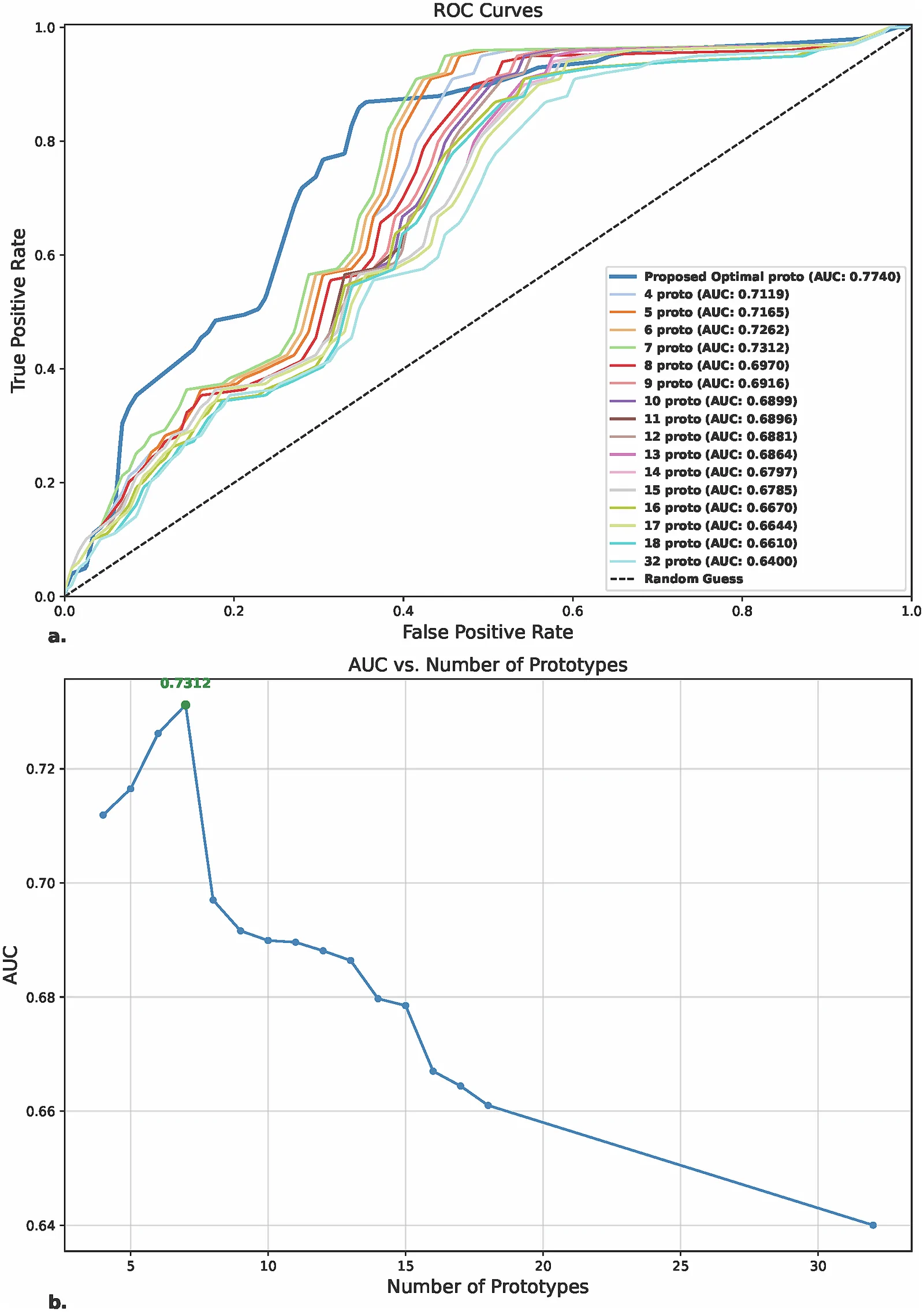
Performance evaluation using the CPTAC external validation cohort. (a) ROC curves evaluating the performance of the linear classifier applying the proposed optimal prototypes versus predefined prototypes. The classifier utilizing the proposed clustering approach obtained the highest AUC (0.7740), indicating its enhanced generalization capability while using unseen datasets. (b) AUC versus cluster numbers in predefined clusters from k-means. Whereas the highest AUC was achieved with seven prespecified clusters, performance consistently declined with an increasing number of clusters, reaffirming the potential issue of over-clustering and the robustness of our proposed approach.

The classifier trained on the TCGA dataset and tested on an unseen GB-UK dataset (Table 4) showed that the classification model utilizing six optimal prototypes obtained the highest AUC of 0.7870 with specificity of 0.660, sensitivity of 0.900, and F1-score of 0.6546. It also surpassed all classifiers using predefined clustering settings (Table 4 and Fig. 7a). Among predefined setting, the classification model based on seven prototypes reached achieved the highest performance (AUC of 0.7551) and after this peak point, performance declined as the number of predefined clusters increased. Moreover, as cluster numbers increased to 18 and 32, the classifier's performance noticeably diminished (Fig. 7b). Subsequently, when using 18 and 32 clusters, the AUC dropped to 0.690 and 0.6350, respectively, and specificity decreased to its lowest values of 0.36 and 0.28, despite maintaining sensitivity at 1. Consequently, the high sensitivity in several k-means-based models reflects a tendency toward over-prediction of EGFR-amplified, resulting in low specificity and indicating poor true-negative discrimination.

**Fig. 7:**
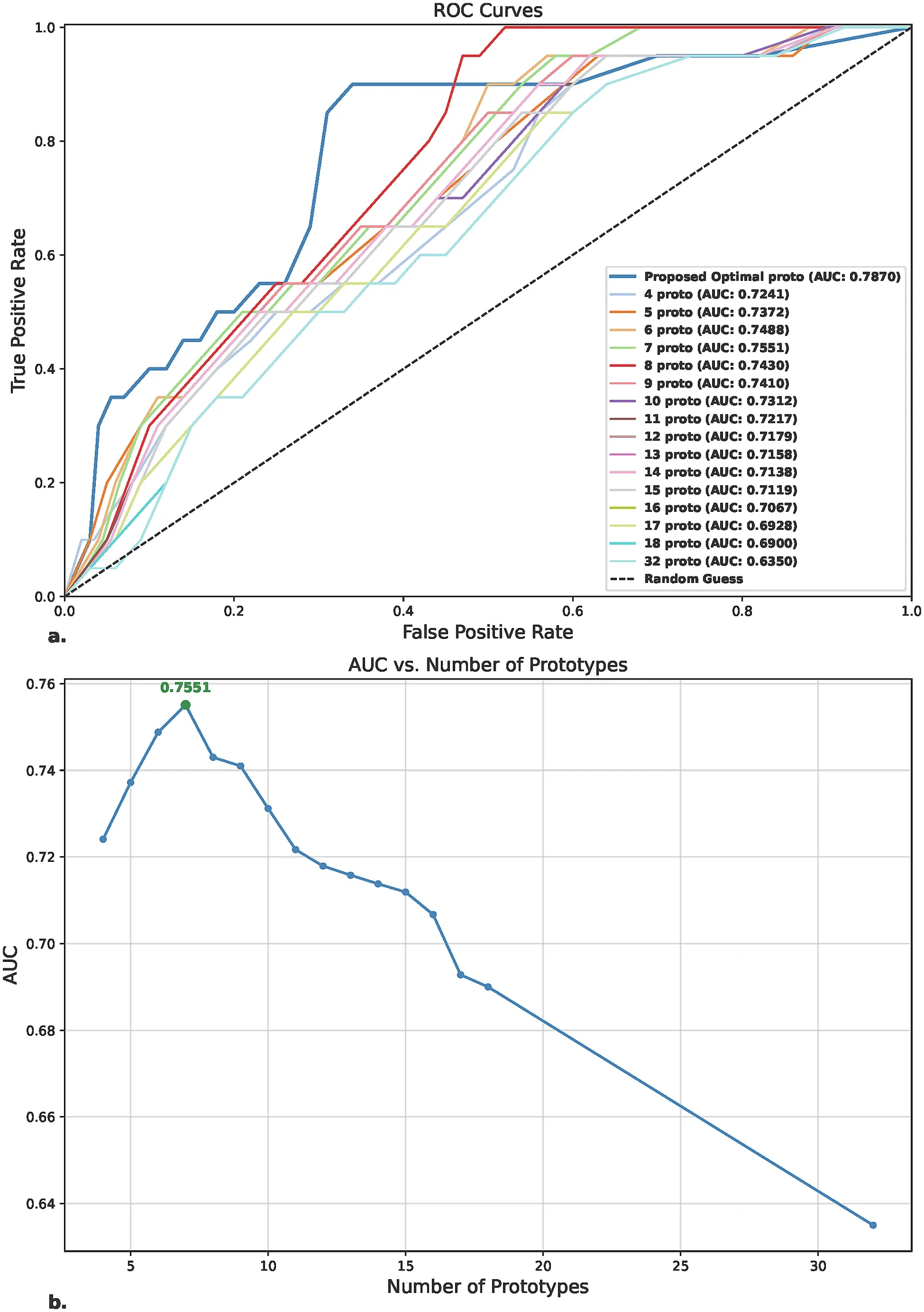
Performance evaluation using the GB-UK external validation cohort. (a) ROC curves compare the performance of the MLP classifier using the proposed optimal prototypes versus prespecified prototypes. The MLP classifier utilizing the proposed clustering approach yielded the highest AUC (0.7870), surpassing all fixed clustering settings, demonstrating its robust generalizability on external datasets. (b) AUC versus cluster numbers in fixed cluster settings. The AUC peaked at seven clusters (AUC: 0.7551), followed by a consistent decline as the cluster numbers increased. The results demonstrate the effectiveness of using optimal prototypes on classification performance and the potential over-clustering issues associated with predefined clusters based on k-means.

Overall, these findings confirm that the optimal prototypes derived from the proposed model offer generalization, which enhances classifiers' discriminative capability on unseen data. Noting that the external cohorts evaluated here are relatively small and limited in diversity, further analysis in broader clinical settings is suggested.

### Optimal clustering outperforms MIL approaches in GBM-EGFR amplification prediction

Given the clinical significance of a reliable GBM-EGFR amplification prediction, we compared the classification performance achieved by our proposed automatic clustering approach with established MIL models. The primary objective of this comparison is to evaluate whether our proposed approach can offer improvements and robustness in EGFR amplification prediction compared to MIL models. Table 5 summarizes the 5-fold cross-validation results using TCGA dataset, under three different setups, including classification results using our proposed optimal clusters, three state-of-the-art MIL models (i.e., CLAM,^29^ TransMIL^40^ and DTFD,^41^ and ACMIL^42^) using UNI feature encoder^31^ and also the same three MIL models of the previously published study^14^ using ResNet feature encoder.^46^ Table 5 also includes the results of a transformer-based model^24^ using different multi-institutional datasets (TCGA/BWH/UPenn). The classifier using optimal prototypes, trained and tested on the TCGA dataset, achieved the highest AUC (0.8087 ± 0.0153). In contrast, MIL models using the same TCGA dataset achieved lower AUC: CLAM (0.763 ± 0.085), DTFD (0.721 ± 0.062), and TransMIL (0.690 ± 0.161) and higher specificity values. For instance, DTFD achieved the highest specificity of 0.961 ± 0.056. Among the MIL models, ACMIL achieved the second highest AUC (0.7585 ± 0.0270) with a sensitivity of 0.9900 ± 0.0223 and the lowest specificity of 0.4883 ± 0.1137. The highest specificity of the MIL models could not elevate the overall classification performance and led to lower AUC and sensitivity in most EGFR cases, indicating the classifier's bias toward EGFR-amplified cases. The classification results of the study,^23^ which applied the same MIL models (reported an AUC of 0.736 ± 0.181, 0.706 ± 0.076, and 0.688 ± 0.172 for CLAM, DTFD, and TransMIL, respectively), and another study^24^ using a transformer-based model with an AUC of 0.71 ± 0.031 remained below the performance of our proposed approach. Consequently, the results suggest that the classification model using our proposed prototype strategy outperformed MIL-based models, enhancing the EGFR prediction efficiency and yielding higher AUC and sensitivity while maintaining an effective balance with specificity.

**Table 5:** 5-fold cross-validation performance (Mean ± SD) evaluation using MIL models.

Dataset	Model type	Feature encoder	Ref	Model	AUC	Specificity	Sensitivity
TCGA	Proposed prototype-based	UNI	This study	MLP	**0.8087** **± 0.0153**	0.7380 ± 0.1181	0.9385 ± 0.0422
				CLAM	**0.7630 ± 0.085**	0.741 ± 0.160	0.786 ± 0.128
TCGA	MIL-based	UNI	This study	DTFD	0.721 ± 0.062	**0.961 ± 0.056**	0.558 ± 0.103
				TransMIL	0.69 ± 0.161	0.088 ± 0.018	**1.0000 ± 0.000**
				ACMIL	0.7585 ± 0.0270	0.4883 ± 0.1137	0.9900 ± 0.0223
TCGA	MIL-based	ResNet	14	CLAM	**0.736 ± 0.181**	0.545 ± 0.351	**0.741 ± 0.194**
				DTFD	0.706 ± 0.076	**0.959 ± 0.067**	0.512 ± 0.113
				TransMIL	0.688 ± 0.172	0.627 ± 0.419	0.548 ± 0.604
TCGA/BWH/UPenn	MIL-based Vision Transformers	N/A	15	Transformer based-CHARM	0.71 ± 0.031	N/A	N/A

In **Table 5**, boldface highlights the proposed model’s highest AUC and the highest values obtained among the MIL-based competing models for the corresponding metrics. The same convention is applied in **Table 6** for the external-cohort comparisons. It is interpreted in the Results section.

### Generalization capability of optimal-prototype-based classification over MIL models on external cohorts

We benchmarked classification performance beyond cross-validation settings. We conducted an external validation comparing optimal prototype models with MIL-based models (CLAM, TransMIL, DTFD, and ACMIL)^42^ utilizing two unseen cohorts: CPTAC and GB-UK (Table 6). All classifiers were trained on the TCGA dataset and tested using whole external datasets, separately. The classifier using optimal clusters and tested on the CPTAC outperformed all other models with an AUC of 0.7740, specificity of 0.6525, sensitivity of 0.8585, and F1-score of 0.7555. In contrast, MIL models did not show significant performance. The CLAM obtained the highest AUC (0.7698) among all MIL-based models tested on CPTAC, with remarkably low specificity (0.200), indicating the model's bias toward the prediction of positive cases (sensitivity: 0.800) at the cost of high prediction of FPs. The ACMIL obtained the second highest AUC of 0.7603 and the low specificity of 0.4915. TransMIL and DTFD exhibited low performance, with AUCs of 0.7581 and 0.7537, respectively, alongside notably lower F1-scores of 0.5192 and 0.5121.

**Table 6:** Performance evaluation between optimal prototype-based classifiers and MIL models using unseen cohorts.

Test dataset	Model type	Model	AUC	Specificity	Sensitivity	F1-score
CPTAC	Proposed prototype-based	MLP	**0.7740**	**0.6525**	**0.8585**	**0.7555**
	MIL-based	CLAM	**0.7698**	0.2000	0.8000	0.6154
		TransMIL	0.7580	0.5339	0.5454	0.5192
		DTFD	0.7537	0.5339	0.53535	0.51208
		ACMIL	0.7603	0.4915	0.8282	0.6804
GB-UK	Proposed prototype-based	MLP	**0.7870**	0.6600	**0.9000**	**0.6546**
	MIL-based	CLAM	**0.7605**	**0.6800**	0.800	0.6153
		TransMIL	0.7380	0.020	0.8500	0.3953
		DTFD	0.7348	0.5000	0.5353	0.5023
		ACMIL	0.7520	0.6000	0.9000	0.6206

In **Table 5**, boldface highlights the proposed model’s highest AUC and the highest values obtained among the MIL-based competing models for the corresponding metrics. The same convention is applied in **Table 6** for the external-cohort comparisons. It is interpreted in the Results section.

Comparing the models tested on the GB-UK external dataset, we observed a significant gap between prototype-based models and MILs. The classifier using optimal-prototype approach outperformed all other models. Among MIL models, CLAM achieved higher classification performance with an AUC of 0.7870, specificity of 0.660, sensitivity of 0.9000, and F1-score of 0.6154. The ACMIL gained the second highest AUC of 0.7520 with the highest sensitivity of 0.9000. The TransMIL obtained relatively low performance with an AUC of 0.738 and notably low specificity (0.020). Similarly, DTFD resulted in an AUC of 0.7348 and reduced sensitivity (0.5353).

### Ablation study

#### Evaluation of how various dimensional features impact classification performance

To validate the selected latent dimension for the proposed framework, we conducted a sensitivity analysis by comparing various dimensions against our proposed 512-dimensional latent features. This evaluation includes both the discovery of prototypes and the performance of downstream classification (see Table 7). The classification results are based on the TCGA dataset using the same 5-fold cross-validation that was used in this study. Using full 1024-dimensional features without applying VAE to compress the features achieved seven prototypes and resulted in a moderate decline in EGGFR classification performance, with AUC of 0.7972 ± 0.0194, sensitivity of 0.8582 ± 0.0620, specificity of 0.5953 ± 0.1498, and F1-score of 0.8302 ± 0.0626. Hence, it shows a 1.42% relative reduction in AUC. Moreover, its computational cost (21 h) is substantially higher than that of our proposed 512-dimensional features (10 h).

**Table 7:** Performance assessment using different dimensional features.

Metrics (mean ± SD)	512D (Current study)	1024D (Without VAE)	256D	128D
AUC	0.8087 ± 0.0153	0.7972 ± 0.0194	0.6426 ± 0.0337	0.5968 ± 0.0519
Sensitivity	0.9385 ± 0.0422	0.8582 ± 0.0620	0.9795 ± 0.0281	0.9589 ± 0.0655
Specificity	0.7380 ± 0.1181	0.5953 ± 0.1498	0.2264 ± 0.2039	0.1823 ± 0.2327
F1-score	0.9066 ± 0.0326	0.8302 ± 0.0626	0.8047 ± 0.1194	0.7825 ± 0.124
Cost (h)	10	**21**	8	7.5

In **Table 7**, boldface is additionally used to draw attention to notable comparative values; specifically, the bold training time of 21 h for the 1024-dimensional configuration indicates its substantially greater computational cost relative to the proposed 512-dimensional configuration (10 h) and therefore represents a disadvantage rather than superior performance. It is interpreted in the Results section.

Applying 256- and 128-dimensional representations reduced the number of prototypes to 4. Moreover, due to differences in the applied 256- and 128-dimensional representations, the retained prototypes are not identical across the two dimensions. Both dimensions showed a more severe decline in performance metrics. The AUC of the classification model utilizing 256-dimensional latent features declined to 0.6426 ± 0.0337, indicating a 20.5% performance reduction. More importantly, specificity decreased by 69.32%. Furthermore, the AUC of the classification model trained and tested on the 128-dimensional compressed features fell to 5968 ± 0.0519, demonstrating a 26.20% reduction compared to the classification model using our proposed 512-dimensional features. Utilizing both 256- and 128-dimensional features resulted in an imbalance of sensitivity and specificity metrics, with sensitivity values of 0.9795 ± 0.0281 and 0.9589 ± 0.0655, and specificity values of 0.2264 ± 0.2039 and 0.1823 ± 0.2327 for the 256- and 128-dimensional configurations, respectively. The computational cost was moderately reduced by employing 256- and 128-dimensional features, owing to the smaller pooled feature matrix. The classifier employing the proposed 512-dimensional configuration attained the best overall metrics within a moderate computational expense (10 h), thereby supporting the adoption of the proposed VAE-based feature compression within the framework.

#### DBGMM ablation experiments

α is the DP concentration parameter and controls how sparse the posterior weight distribution can become. Although it does not directly select the prototypes, it shapes the initial raw weight vectors that will go through the elbow thresholding procedure. To evaluate the practical impact of the concentration parameter (α), we compared the classification performance across models utilizing various α values (0.01, 1, and 5), with the results of our proposed model using α=0.05. This sensitivity analysis was conducted on the TCGA dataset using 5-fold cross-validation, fixing all parameters and varying only α. The comparison results (Table 8) presents that the final retained prototype set was identical (six prototypes) using α=0.05and0.01, and therefore gained the same AUC of 0.8087 ± 0.0153 suggesting stability in the neighborhood of the selected values. As the value of α increased to 1 and 5, the corresponding retained prototype count decreased to 8 at α=1 and 15 at α=5, and showed reduced AUC of 0.7206 ± 0.0106 and 0.6944 ± 0.0290, respectively. These outcomes support our selected small and sparsity-inducing concentration parameter.

**Table 8:** Impact of α on retained prototypes and classification AUC.

α	Final retained prototypes	AUC (mean ± SD)
0.01	6	0.8087 ± 0.0153
0.05^a^	6	0.8087 ± 0.0153
1	8	0.7206 ± 0.0106
5	15	0.6944 ± 0.0290

a In this study, α was assigned to 0.05.

Because the final threshold is determined by knee-detection applied to the cumulative weights using the elbow method, we applied Gaussian smoothing only to moderately smooth the weight curves before the elbow method. To investigate the impact of c on the classification results and the final retained prototypes, we used the same hyperparameters as the proposed model and subsequently selected various values of c on the TCGA dataset using 5-fold cross-validation (Table 9). The final retained prototypes obtained by the Elbow method and the AUC results (0.8087 ± 0.0153) were unchanged with $c=\left\{136\right\}$, confirming that the selected scale contributes only to mild denoising without altering the underlying structural threshold. In contrast, larger c values (10 and 30) showed decreased AUC with a high number of prototypes (7 prototypes for c=10 and 23 for c=30) suggesting that larger c spreads the weights far apart, which can introduce an arbitrary threshold and might dilute the Elbow method's ability to find the actual threshold.

**Table 9:** Impact of con retained prototypes and classification AUC.

c	Retained prototypes	AUC (mean ± SD)
1^a^	6	0.8087 ± 0.0153
3 and 6	6	0.8087 ± 0.0153
10	7	0.7862 ± 0.0081
30	23	0.7510 ± 0.0105

a In this study, c was assigned to 1.

#### Ablation analysis of the GB-UK external cohort using different imbalanced-aware loss functions

The TCGA training cohort contained a higher proportion of EGFR-amplified cases (217) than of EGFR-non-amplified cases (112), whereas the GB-UK external cohort had the opposite class distribution, comprising 20 EGFR-amplified and 50 EGFR-non-amplified WSIs. To investigate whether imbalance-aware training could improve the sensitivity–specificity balance on this external cohort, we retrained the TCGA classifier using focal loss and class-weighted cross-entropy and then evaluated the resulting models on the GB-UK cohort. Table 10 presents a performance comparison of the cross-entropy loss, used as the default loss function in the current study, and the imbalance-aware focal and class-weighted cross-entropy loss functions. With the default cross-entropy, the model obtained a sensitivity of 0.90 and specificity of 0.66, corresponding to 2 FNs and 17 FPs. Focal loss reduced FPs to 14, increasing specificity to 0.72, balanced accuracy to 0.81, and F1-score to 0.692 while preserving sensitivity at 0.90. Class-weighted cross-entropy reduced FPs to 12. It also increased specificity to 0.76, balanced accuracy to 0.83, and F1-score to 0.720, whereas sensitivity remained at 0.90. Moreover, AUC remained at 0.787 across all three loss functions indicating that the imbalance-aware loss functions improved the classification balance at the selected operating threshold rather than the overall ranking of cases.

**Table 10:** Performance evaluation between the application of cross-entropy loss and imbalance-aware loss functions on classification results of our proposed model training on TCGA and testing on unseen GB-UK cohort.

Loss function	AUC	Specificity	Sensitivity	Balanced accuracy	F1-score	FP	FN
aCross-entropy	0.787	0.660	0.900	0.780	0.655	17	2
Focal loss	0.787	0.720	0.900	0.810	0.692	14	2
Class-weighted cross-entropy	0.787	0.760	0.900	0.830	0.720	12	2

a Our proposed model is trained using cross-entropy loss.

#### Performance analysis over a different feature encoder

To further investigate the proposed model's performance under a different feature encoder, we adopted Phikon-v2,^47^ a foundation model pre-trained on 450 million histology images. Following the same segmentation and tiling preprocessing pipeline, WSIs were divided into 224 × 224 patches, and Phikon-v2 was applied to extract 1024-dimensional feature embeddings. These features were then compressed to a 512-dimensional representation using the VAE, followed by DBGMM to derive the optimal prototypes for the TCGA cohort, resulting in seven prototypes. 5-fold cross-validation was performed using the same folds applied to the UNI feature embeddings, and classification results were compared against models using predefined prototype numbers. Table 11 presents the 5-fold cross-validation results on the TCGA cohort using the Phikon-v2 feature encoder. The proposed automatic prototype learning approach, which identified seven prototypes, outperformed both predefined prototype settings (8 and 16) across all four metrics, achieving the highest AUC (0.8294 ± 0.0205), specificity (0.7820 ± 0.0943), sensitivity (0.9478 ± 0.0514), and F1-score (0.9226 ± 0.0381). Performance also declined as the predefined prototype number increased from 8 to 16, suggesting that an excessive number of prototypes may introduce redundancy or noise into the classification process. These results are consistent with those obtained using the UNI encoder, further confirming that the proposed automatic prototype learning strategy generalizes effectively across different feature encoders.

**Table 11:** 5-fold cross-validation classification results of TCGA cohort (using Phikon-v2 feature encoder) based on clustering methods and given prototype numbers.

Clustering	Prototype	AUC (Mean ± SD)	Specificity (Mean ± SD)	Sensitivity (Mean ± SD)	F1-score (Mean ± SD)
Proposed automatic prototype learning	**7**	**0.8294** **± 0.0205**	**0.7820** **± 0.0943**	**0.9478** **± 0.0514**	**0.9226** **± 0.0381**
Predefined number of prototypes	8	0.7703 ± 0.0383	0.7153 ± 0.1304	0.7352 ± 0.3310	0.7410 ± 0.2748
	16	0.7355 ± 0.0161	0.5183 ± 0.3007	0.6261 ± 0.2406	0.6481 ± 0.1930

In **Table 11**, boldface highlights the performance of the proposed approach.

## Discussion

EGFR amplification plays a pivotal role in the diagnosis of GBM and in therapeutic decision-making.

Current molecular diagnostic standards are costly and resource-intensive and are not accessible in all clinical environments. These challenges delay molecular testing, which hinders patients' treatment timelines. Therefore, there is a crucial need to develop a pathology-based computational model that provides a fast, precise, and widely accessible approach for predicting EGFR amplification from WSIs. In this study, we demonstrated that an automatic interpretable prototype-learning approach predicts GBM-EGFR amplification with high performance across internal and external validations. Although the external cohorts evaluated here are relatively limited in size. It also demonstrated a robust ability to link the prototypes to neuropathologists' recognizable tissue patterns.

A main challenge with prediction models using WSIs is to capture all morphological features of the tissue in a way that supports precise classification and interpretability. In this regard, MIL methods have achieved outstanding performance in classifying WSIs, particularly for predicting EGFR amplification. However, they rely only on slide-level labels and attention-based selected tissue patches, failing to capture global morphological tissue structures.

On the other hand, conventional unsupervised models rely on predefined, arbitrary cluster numbers, which can lead to over- or under-clustering. Over-clustering results from over-fragmenting the tissue's morphological features, whereas under-clustering results from restricted merging of diagnostically distinct tissue morphologies.

To address the challenges, we proposed an automatic prototype-learning framework for GBM-EGFR prediction that learns data-driven tissue morphological prototypes without requiring a predefined number of clusters or manual annotations.

The superior performance of the proposed model stems from its two major components. First, the application of VAE reduces feature sparsity while preserving informative morphological structure. Classification results using the 1024-dimensional features (without applying VAE) yielded a moderate AUC of 0.7972 ± 0.0194, but with lower specificity and sensitivity than those achieved by the model using a 512-dimensional embedding. Hence, it indicates that high-dimensional features increase sparsity. Furthermore, as the pairwise distances between patches become more concentrated with increasing dimensionality, clustering methods become less effective at distinguishing different tissue morphologies.^48^ Conversely, over-compressing the data to 256 and 128 dimensions resulted in a notably different and more severe failure mode. Instead of a moderate, consistent decline, the classification performance specifically deteriorated in discriminative ability, with AUC scores of 0.6426 and 0.5968, respectively. Therefore, results demonstrate that 512 dimensions represents the optimal trade-off between preserving discriminative morphological information and mitigating the instability associated with high-dimensional clustering, achieving the best classification performance at moderate computational cost.

Moreover, the DBGMM enables the model to more effectively explore the underlying data structure and determine the optimal clusters without relying on arbitrary, predefined cluster numbers.

Across internal and external validations, the proposed approach presented a more balanced sensitivity–specificity than the predefined clustering approach. As a result, it suggests that the automatic determination of morphological prototypes overcomes the over- and under-clustering effects that reduce discrimination between EGFR-amplified and EGFR-non-amplified cases.

Benchmarks against MIL models indicated that the proposed framework achieved higher classification performance. Although MIL models achieved competitive performance, they consistently showed imbalanced sensitivity and specificity on unseen validation cohorts, due to their attention-based aggregation strategy, which can lead to focus on non-discriminative tissue patterns, hindering their ability to overcome noisy instances and generalize across various cohorts.^28^, ^29^, ^49^, ^50^

A key finding of our model's quantitative prototype analysis based on Cohen's d revealed that prototype C4 exhibited the strongest ability to discriminate EGFR-amplified from EGFR-non-amplified cases, followed by C5, C2, and C3 prototypes. Neuropathology review indicated that C3 and C4 prototypes were characterized by low-cell-density regions with either minimally infiltrated or subtly abnormal brain tissue. As a result, it suggests that the learned prototypes were not only effective in the prediction model but also could present morphologically interpretable patterns. An experimental study using genetically engineered mouse models^4^ has shown that EGFR amplification or mutant EGFR signaling causes diffuse parenchymal invasion, tumor cell dispersal along neural structures, and invasion at tumor margins rather than bulk hypercellularity. Furthermore, a similar study^51^ on human GBM demonstrated that wild-type EGFR amplification enhances tumor growth without the need for angiogenic remodeling. These growth patterns are compatible with regions of deceptively preserved or only inconspicuously abnormal parenchyma surrounding invasive edges. A xenograft modeling study also demonstrated that patient-derived GBM cells with EGFR amplification transplanted into the brain and flanks of nude mice led to formation of invasive tumors that retained EGFR amplification^52^; in GBM slice models, inhibition of EGFR by gefitinib led to a reduction in GBM cell invasion for EGFR amplified tumors.^53^

These prior studies, showing EGFR-associated diffuse invasion, suggest the biological patterns that are highlighted by our infiltration-associated morphology findings. Therefore, the convergence of known tumor biology, neuropathological interpretation, quantification of the prototypes' effect size, and the automatic prototype learning provide a convincing explanation for why these prototypes play a significant role in the model's decision-making for EGFR-amplified cases.

From the pathology informatics standpoint, the results suggest that the prototype learning's contribution extends beyond solely improving the efficiency of the prediction model. As the framework represents WSIs as semantically meaningful tissue prototypes, it provides a more transparent and interpretable basis for analysis than relying only on a few selected patches. Therefore, this interpretable concept is particularly relevant to the digital pathology workflow, where molecular testing is crucial yet constrained by high cost, limited accessibility, and time-consuming nature. A morphology-driven AI prediction framework capable of identifying optimal prototypes of H&E-stained WSIs could be a strong assistive tool for molecular testing by facilitating case prioritization and providing rapid, early decision-support, especially in resource-constrained settings. Nevertheless, this approach should be considered complementary to molecular testing rather than a diagnostic replacement.

### Limitations

Despite the promising outcomes of the proposed framework, it is important to acknowledge the study's limitations to inform future development. This study focused solely on GBM-EGFR amplification, and the generalizability of the framework to EGFR mutations (e.g., EGFRvIII) and broader GBM biomarkers (e.g., IDH, MGMT, and TERT^54^) remains to be evaluated.

Although the same preprocessing methods, including tissue segmentation, WSI tiling, and feature extraction, were applied across all three cohorts (i.e., TCGA, CPTAC, and GB-UK datasets), variations in fixation, staining intensity, tissue quality, and scanners may have introduced cohort-specific domain shifts. Hence, the use of an identical preprocessing pipeline reduces procedural variability; however, it cannot overcome preanalytical or scanner-related differences. Moreover, the retrospective inclusion of cases with available molecular annotations and WSIs of adequate quality may introduce selection bias and may not fully reflect the broader population encountered in routine clinical practice. Demographic and clinical factors, such as age, sex, ethnicity, institution, treatment history, and tumor sampling characteristics, were not formally adjusted. Therefore, it may represent residual confounders. In addition, the different class distributions and the relatively small size of external cohorts may affect the stability of sensitivity and specificity predictions.

Because EGFR-amplified cases dominated TCGA training cohort, it may lead the model to learn toward positive predictions when testing on the external GB-UK cohort, which includes the opposite class distribution. Consequently being biased toward positive cases. The model predicted 18 out of 20 EGFR-amplified cases in the GB-UK dataset, achieving high sensitivity (0.90), however, it misclassified 17 out of 50 EGFR non-amplified cases, resulting in lower specificity (0.66). Whereas sensitivity and specificity were calculated based on their respective classes the FP burden in the GB-UK cohort, which has predominantly EGFR-amplified cases, reduced the prevalence-dependent metrics such as F1-score. The imbalance-aware loss functions (focal loss and class-weighted cross-entropy) partially mitigated this effect, reducing FPs and increasing balanced accuracy from 0.78 to 0.81 and 0.83, while maintaining sensitivity at 0.90. Nonetheless, the observed performance might be influenced by differences in staining, tissue preprocessing, scanner settings, and other biological factors. Therefore, validation on broader, multi-institutional external cohorts is required to evaluate generalizability.

Finally, these findings suggest that the proposed framework has potential to yield interpretable and generalizable GBM-EGFR predictions, though broader validation is required to establish its clinical robustness. Hence, by effectively capturing tissue morphology, this approach can enhance digital pathology workflows and assist clinical decision-making.

## CRediT authorship contribution statement

H.D.M: conceived the idea of this work, performed data curation, developed the methodology and software, provided resources, created visualizations, conducted formal analysis, supervised the project, and wrote the original draft, including review and editing. I·N: contributed to the review and editing, provided clinical investigation and contributed to the collection of the UK-Brain WSI Data. C.C: contributed to the review, editing and Investigation. M.F: contributed to the review and clinical investigation. A.D·I: Contributed to the review, editing, and provided clinical investigation. S.L: provided supervision, resources, review, and editing and contributed to the investigation. All authors reviewed and approved the final manuscript.

## Ethics declaration

Images of the GB-UK dataset were obtained with Ethical approval from the UK Brain Archive Information Network (BRAIN UK) and the Research Ethics Committee South Central Hampshire B (REC reference number 14/SC/0098). Written informed consent was obtained from all patients in accordance with BRAIN UK protocols.^8^, ^55^

## Code availability

The code used to develop and evaluate our model will be made publicly available on GitHub upon acceptance of the manuscript.

## Declaration of generative AI and AI-assisted technologies in the writing process

During the preparation of this work, the authors used ChatGPT and Grammarly to improve the manuscript's readability. After using these tools, the authors reviewed and edited the content as needed and take full responsibility for the content of the published article.

## Funding

This research was supported by the Commonwealth through an Australian Government Research Training Program [https://doi.org/10.82133/C42F-K220] to H.D.M. The generation and curation of the GB-UK dataset were supported by funding to I·N from a 10.13039/100005622National Institute of Health Research Clinical Lectureship, the 10.13039/501100000765University College London Biomedical Research Centre, and the 10.13039/501100000691Academy of Medical Sciences (grant number: SGL026\1035). NHMRC Ideas (ref no. GNT2020035) Computational Analysis and AI in Brain Tumor Imaging: Toward the Augmented Diagnostics of the Future to A.D, S.L and C·C.

## Declaration of competing interest

The authors declare that they have no known competing financial interests or personal relationships that could have appeared to influence the work reported in this article.

## Data Availability

The TCGA and CPTAC WSI datasets are publicly available through the National Cancer Institute Cancer Research Data Commons (CRDC) at https://www.cancer.gov/ccg/. The GB-UK dataset is not publicly accessible due to privacy and ethical restrictions; however, it may be made available upon reasonable request, subject to appropriate institutional review board approval.
